# Diabetes, HIV and other health determinants associated with absenteeism among formal sector workers in Namibia

**DOI:** 10.1186/1471-2458-12-44

**Published:** 2012-01-18

**Authors:** Leonor Guariguata, Ingrid de Beer, Rina Hough, Els Bindels, Delia Weimers-Maasdorp, Frank G Feeley, Tobias F Rinke de Wit

**Affiliations:** 1 PharmAccess Foundation, Trinity Building C, Pietersbergweg 17, 1105, BM Amsterdam, Zuidoost, the Netherlands; 2 PharmAccess Foundation, P.O. Box 9895, Windhoek, Namibia; 3 Boston University School of Public Health, 715 Albany Street, Talbot Building, Boston, MA 02118, USA; 4 Amsterdam Institute for Global Health and Development (AIGHD), Academic Medical Center, University of Amsterdam, Amsterdam, the Netherlands

**Keywords:** Absenteeism, Namibia, Work force, Sub-Saharan Africa, Non-communicable disease, HIV

## Abstract

**Background:**

As countries in sub-Saharan Africa develop their economies, it is important to understand the health of employees and its impact on productivity and absenteeism. While previous studies have assessed the impact of single conditions on absenteeism, the current study evaluates multiple health factors associated with absenteeism in a large worker population across several sectors in Namibia.

**Methods:**

From March 2009 to June 2010, PharmAccess Namibia conducted a series of cross-sectional surveys of 7,666 employees in 7 sectors of industry in Namibia. These included a self-reported health questionnaire and biomedical screenings for certain infectious diseases and non-communicable disease (NCD) risk factors. Data were collected on demographics, absenteeism over a 90-day period, smoking behavior, alcohol use, hemoglobin, blood pressure, blood glucose, cholesterol, waist circumference, body mass index (BMI), HIV status, and presence of hepatitis B antigens and syphilis antibodies. The associations of these factors to absenteeism were ascertained using negative binomial regression.

**Results:**

Controlling for demographic and job-related factors, high blood glucose and diabetes had the largest effect on absenteeism (IRR: 3.67, 95%CI: 2.06-6.55). This was followed by anemia (IRR: 1.59, 95%CI: 1.17-2.18) and being HIV positive (IRR: 1.47; 95%CI: 1.12-1.95). In addition, working in the fishing or services sectors was associated with an increased incidence of sick days (IRR: 1.53, 95%CI: 1.23-1.90; and IRR: 1.70, 95%CI: 1.32-2.20 respectively). The highest prevalence of diabetes was in the services sector (3.6%, 95%CI:-2.5-4.7). The highest prevalence of HIV was found in the fishing sector (14.3%, 95%CI: 10.1-18.5).

**Conclusion:**

Both NCD risk factors and infectious diseases are associated with increased rates of short-term absenteeism of formal sector employees in Namibia. Programs to manage these conditions could help employers avoid costs associated with absenteeism. These programs could include basic health care insurance including regular wellness screenings.

## Background

Absenteeism due to health factors is mainly studied in high-income countries. In many of these countries, social mechanisms such as health insurance and national health systems are in place to support and care for employees who are faced with illness. This is not the case for many low- and middle-income countries where employees must pay for care directly out-of-pocket. As a result, absenteeism in the workforce in low- and middle-income countries may place a greater burden on employees who do not have access to health care, as well as to employers who must bear the burden of lost productivity.

As low and middle-income countries work to compete in the global market, the health of their workforce is increasingly vital to enhance productivity. For many sub-Saharan African countries, infectious diseases like HIV/AIDS are still having a serious impact despite the large-scale campaigns for prevention, care and anti-retroviral therapy (ART) [1]. Non-communicable diseases (NCDs) are playing an important role, as changing diets and sedentary habits are adopted, particularly in urban settings [2]. Conditions like cardiovascular disease and diabetes are increasingly affecting countries in economic transition and are expected to increase dramatically over the next 20 years [3,4]. Despite these trends, funding to prevent and manage NCDs is significantly behind that of communicable disease [5]. This is remarkable in light of the fact that cost-effective and cost-saving interventions have been shown to prevent NCDs in developing countries [6] and are adaptable to the workplace.

For many sub-Saharan African countries, unlike high-income countries which have many more social support mechanisms in place, individuals pay out-of-pocket for their own care [7-9] which creates a significant barrier to access. As a result, individuals in the workforce faced with the high cost of managing disease have few options. There is some evidence of employers filling this gap by providing basic health care in the workplace [10]. Employers must balance the provision of health services and health insurance to their workers while maintaining profitability, taking into account the double burden of infectious and non-infectious chronic diseases. If the worker is not paid for some or all of the sick time, household income falls at the same time that medical care costs may increase. Furthermore, sickness related absenteeism is an indicator of the prevalence and severity of health conditions in the work force, an indicator that is not collected through the health system. Loss of productivity due to health-related absenteeism remains a barrier to productivity and thus to economic development. Gathering information regarding employee health can help employers make informed decisions to keep their workforce healthy and productive and reduce absenteeism as well as improving access to care for individuals.

Current research into absenteeism and its determinants, concentrated in high-income countries, has found that with regards to health, NCDs are contributing significantly to increased sick leave [11-14]. However, similar research among employees in sub-Saharan Africa has focused primarily on the contribution of infectious disease and in particular HIV/AIDS [15-17]. In addition, since HIV/AIDS has largely affected those of working age, it is important to understand the impact of being infected on employees and the workplace. In a previous study we reported on the HIV prevalence amongst the workforce in Namibia [18]. There is a gap in research looking more generally at the determinants of absenteeism among workers in sub-Saharan Africa. The findings from this paper will provide evidence that may help fill that gap and drive further research in this area.

Namibia represents a country in economic and epidemiologic transition in sub-Saharan Africa. It has one of the region's highest rates of gross-national income per capita at USD 4,210 in 2008, compared to an average of just USD 1,082 for all of sub-Saharan Africa [19]. However, the country is also characterized by one of the highest income disparities in the world [19]. While Namibia posts better than average performance on a number of economic measures when compared to the rest of sub-Saharan Africa [19], it has one of the highest national rates of HIV/AIDS at 13.3% [1]. The government has engaged in a substantial program to provide anti-retroviral therapy at a low cost and improve access to care [1]. This has resulted in almost universal coverage of anti-retroviral therapy. The majority of health services in Namibia are concentrated in a few cities in the north and center of the country [20]. This leaves a portion of the population without easy access to basic care. While the Ministry of Health and Social Services (MOHSS) has developed and implemented a program to improve access to care for more remote regions [20] it can still be a long distance to travel. As a consequence, large employers often provide basic healthcare services on site. In addition, some employers offer (co)payment of healthcare insurances for their employees (and dependents) through one of the existing Namibian medical aid schemes [10]. However, provision of these services is based on the perceived needs of the employee and employer rather than on actual evidence of disease risks and determinants.

This study is based on the results of wellness surveys that were performed at the request of employers from 7 different Namibian industry sectors. These wellness surveys are the most extensive yet in that country. The industry sectors assessed were retail, agriculture, fishing, services, wholesale, tourism and transport. In addition, the survey provides information on the prevalence of certain risk factors and conditions among different industries in the Namibian private sector. The primary goals of this paper are:

1. Describe the demographic and work profile of participants including industry, contract type, job type, age, gender, and the prevalence of risk factors and conditions such as hypertension, diabetes, HIV, hepatitis B, anemia and syphilis in the Namibia formal sector.

2. Describe the associations of the above determinants to self-reported sickness absence.

Through seeking to answer these questions, this paper aims to understand the determinants of absenteeism in the Namibian formal sector in order to improve the provision of healthcare to Namibian workers.

## Methods

From 2009 to 2010, a voluntary cross-sectional wellness survey and health screening of workers was conducted by PharmAccess Foundation at the request of 42 companies across seven industry sectors in Namibia. Participation in the screening and survey was entirely voluntary and strictly confidential. To protect confidentiality, no identifiers were stored with the information gathered and participants gave written informed consent before inclusion.

The survey was performed by two mobile clinics from the Bophelo! Project, a partnership between PharmAccess, the Namibian Business Coalition on AIDS and the Namibia Institute of Pathology. As part of the project, wellness surveys and health screening services are offered to companies based in Namibia along with sensitization and information sessions provided before employee screenings. The mobile clinic visits a company site for as long as required to see all participating employees (up to 32 people per van per day) and each screening visit takes an average of 30 min for pre- test counseling, testing, and post- test counseling sessions. All services are provided by MOHSS-trained testers and counselors, as part of a national task shifting effort. While screening results are being obtained, the participant receives health education and confidential counseling. Participants who tested positive for any condition or have screening results outside of a normal range are encouraged to seek follow-up consultation and are issued a referral letter to local medical services. Only de-identified information was collected from the screenings without any possibility of linking back to the participant. The study was approved by the Ministry of Health and Social Services of Namibia (MOHSS).

A total of 10,701 employees were offered the possibility to participate in the survey across all companies; 8,355 provided written informed consent, of which 100 were excluded from analysis because of pregnancy, as this was considered to be a risk for potential bias for absenteeism. 589 were excluded because of being younger than 20 years of age which could also bias findings on absenteeism. In summary, 7,666 participants were included in this analysis. Participants were asked to recall how many days they had missed work due to sickness in the 90 days prior to administration of the survey. To preserve confidentiality, this measure was based entirely on participant recall and not compared to employer records.

Participants who provided informed consent were asked to complete a questionnaire with demographic (age, sex, marital status, smoking), job status information (contract type, type of position), and self-reported presence of the following conditions: HIV/AIDS, diabetes, and hypertension. In addition, participants who agreed received a medical screening including risk factors (haemoglobin, systolic and diastolic blood pressure and anthropometric markers: height, weight and waist circumference) and prevalence of diseases (HIV status, hepatitis B antigens, syphilis, random blood glucose for diabetes). A finger prick was conducted on all participants to collect a blood sample for the following rapid tests: HIV, syphilis antigen, hepatitis B antigen, haemoglobin and blood glucose. Participants were allowed to refuse any or all of these screenings.

The following assays and methods were used for collecting medical screening information according to manufacturer instructions. Any test with an inconclusive or discordant result was repeated. For HIV testing, the Determine HIV 1/2 Assay by Inverness rapid test strip and Trinity Biotech Uni -Gold HIV 1/2 test kit were used in conjunction for HIV testing, according to Namibian VCT regulations. If the result was discordant between the two tests, a third (tie-breaker) test was performed using the Inverness Clearview Complete HIV 1/2 test kit. Hepatitis B testing was done using the Determine HBsAg Whole Blood Assay rapid test strip (Inverness Medical, USA) was used. The Determine Syphilis TP Whole Blood Assay (Inverness Medical, USA) was used for presence of syphilis antibodies. The Accutrend Plus GCT meters were used to test blood glucose (Roche Molecular Diagnostics, USA). HemoCue Hb 201+ Analyser and HemoCue Hb 201+ microcuvettes (HemoCue, USA) were used to conduct haemoglobin tests. Systolic and diastolic blood pressure was measured in millimetres of mercury (mmHg) by using the MG150f Digital Blood Pressure monitor (Rossmax International Ltd, Taiwan). The measurement was taken three times on each participant and the average of the three readings was used. A blood pressure reading above 140/90 mmHg was considered elevated; above 153/103 mmHg was considered high and referral was made to a medical facility.

Bodyweight was measured with light clothes on, without shoes on an analogue scale and the recorded value was rounded to the nearest 0.5 kg. Height was measured in centimeters using a non-stretch retractable measurement tape to the nearest 0.5 cm. These were used to calculate body mass index according to the formula below.

$$BMI=\frac{weight\left(kg\right)}{height^{2}\left(m^{2}\right)}$$

A BMI of 25-30 was considered overweight; above 30: obese. Waist circumference was measured at the level of the umbilicus in standing position with the hands by the side and the feet together at the end of a normal expiration. A no-stretch retractable tape was used. Measurements were rounded to the nearest 0.1 cm. A waist circumference above 102 cm in men and 88 cm in women was considered above normal and indicative of central obesity.

Only aggregate results were reported back to the employer. Individual screening results were given directly to the participant. Based on individual participant results, the person was advised to make an appointment with a clinician for confirmation and further medical follow-up. In addition, participants with screening results outside the above indicated normal values or who tested positive for any condition were given a letter of referral for a follow-up visit with a clinician including a description of the findings of the screening.

Two variables were created based on a combination of findings from clinical screening and participant responses. Diabetes was classified as known diabetes or those whose random blood glucose was 11.1 mmol/L or higher. Similarly, hypertension was classified as known hypertension, as well as those who were found at screening to have systolic blood pressure above 154 mmHg or diastolic blood pressure above 104 mmHg.

Data were collected and stored using SPSS. All participants received counseling and information on their results and were invited to speak with a trained health professional or to schedule a follow-up with a physician.

### Statistical analysis

All statistical analyses were conducted using R Project for Statistical Computing version 2.10.0 (http://www.r-project.org). 890 participants had missing values for the outcome variable and were thus excluded from univariate and expanded analyses. In many cases, missing data was mostly a result of companies refusing to provide a particular screening to employees. These companies were then dropped from the corresponding part of the analysis. There were no significant differences in demographic and working sector variables for those with missing data compared with those included in the analysis. Descriptive statistics were calculated based on an intent-to-treat model where all those included with sufficient outcome data were analyzed. However, in most cases, results on screenings were not available for some participants as a result of the choice of companies not to offer that screening, rather than the choice of the employee not to undergo the test. Differences between industries for continuous variables were tested using one-way analysis of variance. Chi-squared analysis was used to test differences between industries for categorical variables.

### Outcome of interest

The outcome of interest used for all univariate and expanded analyses was a self-reported measure collected from the survey. Participants were asked to name the number of days they had been absent from work in the previous 90 day period due to health reasons.

### Modeling

All univariate analyses were done using non-parametric tests to allow for the non-normal distribution of the outcome and controlled for by industry. Initially, Poisson's regression models were fit to the data and tested using likelihood ratio tests for over dispersion against a negative binomial models. This test consistently found that the Poisson's model was significantly over-dispersed with respect to the negative binomial model. Thus, negative binomial regression models were used for both the univariate models and expanded models in agreement with published standards for the analysis of absenteeism data [21]. In univariate and expanded analyses, incidence rate ratios (IRR) for sick days were calculated to measure the strength of association for independent variables to the outcome. For all statistical tests, an alpha of 0.05 or less was considered statistically significant. Univariate models used number of sick days in the last 90 days as the outcome and only a single predictor (each risk factor, demographic variable, and condition). Expanded models used number of sick days in the last 90 days as the outcome and assessed the impact of risk factors controlling for industry, gender, age, job status, and contract type.

We constructed dummy variables for the industry variable in order to compare the rates of sickness absence for that variable in regression analyses. The retail sector was chosen as a control group simply as a matter of convenience. The survey was not designed and implemented with a particular pre-selected control group. The retail industry was chosen as the control group post-hoc because it was the largest and because it did not have extreme values for the prevalence of any of the screening-related conditions. The control group was used only for analysis regarding industry sector only.

## Results

### Demographic and job-related information

A description of demographic variables is presented in Table 1 including all 7,666 participants. For all sectors combined, the majority of participants were men (65.3%, 95%CI: 64.2-66.4), and the mean age of participants was 36.3 (95%CI: 36.0-36.5) years. The majority of employees were working in non-administrative labor positions (78.6%, 95%CI: 77.2-79.0) and had permanent contracts (70.5%, 95%CI: 69.4-71.5). Across all companies, the mean number of sick days was 0.92 (95%CI: 0.83-1.01) in the 90 days previous to administration of the survey with a skewing towards few to no sick days. A total of 18.6% of all employees surveyed reported having at least one sick day with 4.3% reporting having missed five or more days. The greatest percentage of absences due to sickness was in the fishing industry (5.6%) (Table 2) followed by the retail and trade industry (5.2%) with the lowest percentage of absences in the agriculture and hotels and restaurants industries (0.6%).

**Table 1:** Distribution of demographic and job-related information study participants

Categorical variables		n	%	95% CI
Sex				

	Male	5,005	65.3%	64.2-66.4
	
	Female	2,661	34.7%	33.6-35.8

Job Type				

	Labour	5,986	78.1%	77.2-79.0
	
	Administration	1,626	21.2%	20.3-22.1

Contract Type				

	Permanent	5,403	70.5%	69.4-71.5
	
	Part-time/Short-term	2,263	29.5%	28.5-30.5

Industry				

	Retail and Trade	2,086	27.2%	26.2-28.2
	
	Agriculture	465	6.1%	5.5-6.6
	
	Fishing	1,869	24.4%	23.4-25.3
	
	Electricity, gas & water	1,140	14.9%	14.1-15.7
	
	Wholesale trade and repair of motor vehicles	1,031	13.4%	12.7-14.2
	
	Hotels and Restaurants	300	3.9%	3.5-4.3
	
	Transport, Storage and Communication	775	10.1%	9.4-10.8

Sick absence				

	Any absence	1,431	18.6%	17.6-19.7
	
	5 or more days absent	334	4.3%	3.9-4.8

**Continuous Variables**		**n**		**95% CI**

Age				

	Mean	36.3		36.0-36.5
			
	Median	35		
			
	Range	20-79		
			
	Missing data	0		

Sick Days				

	Mean	0.92		0.83-1.01
			
	Median	0		
			
	Range	0-90		
			
	Missing data	890		

**Table 2:** Demographic, risk factor, and disease information by industry

Demographic Information												
		Age	Sex		Job type		Contract type		Sick days		Sickness	
**Industry**	**n**	**Mean**	**Male**	**Female**	**Labour**	**Administration**	**Permanent**	**Part-time/Short-term**	**Mean**	**Standard deviation**	**Any absence**	**More than 5 days absent**

Retail and Trade	2,086	33.5	63.0%	37.0%	75.2%	23.9%	70.5%	29.5%	0.7	3.2	5.2%	1.0%

Agriculture	465	37.1	13.7%	38.7%	91.4%	8.2%	90.5%	9.5%	0.5	2.7	0.6%	0.0%

Fishing	1,869	37.6	46.5%	48.0%	93.2%	6.4%	45.9%	54.1%	1.1	4.7	5.6%	1.3%

Electricity, gas & water	1,140	40.0	43.1%	21.1%	61.8%	37.7%	82.8%	17.2%	1.2	4.6	3.6%	1.0%

Wholesale trade and repair of motor vehicles	1,031	36.4	37.8%	23.6%	81.4%	17.8%	81.9%	18.1%	1.0	3.8	3.1%	0.8%

Hotels and Restaurants	300	31.2	6.7%	53.7%	81.0%	18.3%	63.3%	36.7%	0.7	4.0	0.6%	0.2%

Transport, Storage and Communication	775	36.2	29.1%	21.5%	60.0%	38.8%	87.2%	12.8%	0.9	2.7	2.5%	0.6%

**Risk Factors**												

		**Blood Pressure**			**BMI**				**Waist Circumference**		**Smoking behaviour**	

Industry	n	Normal (< 140/90 mm/Hg)	Elevated or High (140/90-153/103 mm/Hg)	High (≥ 154/104 mm/Hg)	Underweight (< 18.5)	Normal (18.5-24.9)	Overweight (25-29.9)	Obese (≥ 30)	Normal	Above limit	Never smoked	Smokes

Retail and Trade	2,086	72.3%	17.4%	10.1%	7.0%	57.9%	21.4%	12.4%	86.2%	13.3%	81.8%	16.1%

Agriculture	465	71.0%	19.8%	8.8%	17.2%	58.7%	13.5%	10.3%	88.0%	11.8%	64.7%	34.8%

Fishing	1,869	67.3%	18.4%	13.9%	3.7%	50.5%	26.6%	18.9%	77.8%	21.7%	85.0%	13.1%

Electricity, gas & water	1,140	71.8%	18.9%	9.1%	4.4%	46.9%	28.9%	18.9%	80.1%	18.9%	82.5%	15.5%

Wholesale trade and repair of motor vehicles	1,031	82.3%	12.0%	5.7%	6.7%	64.6%	18.3%	9.9%	88.2%	11.5%	81.0%	17.4%

Hotels and Restaurants	300	79.3%	13.7%	7.0%	6.7%	48.7%	28.3%	16.3%	79.3%	20.7%	78.76%	19.0%

Transport, Storage and Communication	775	74.8%	15.7%	9.2%	5.4%	50.3%	28.5%	15.4%	82.3%	17.4%	74.6%	24.6%

**Conditions and Diseases**												

Industry	n	Hypertension	Diabetes	Anemia	HIV	Hepatitis B	Syphilis					
					
Retail and Trade	2,086	14.0%	0.9%	6.5%	8.3%	7.3%	0.8%					
					
Agriculture	465	12.9%	0.9%	3.9%	5.6%	8.0%	3.9%					
					
Fishing	1,869	21.7%	1.8%	10.2%	14.3%	4.2%	0.2%					
					
Electricity, gas & water	1,140	21.1%	3.6%	4.7%	8.2%	7.6%	1.6%					
					
Wholesale trade and repair of motor vehicles	1,031	12.7%	1.6%	2.9%	7.9%	9.2%	1.7%					
					
Hotels and Restaurants	300	10.7%	0.7%	4.7%	5.7%	5.3%	0.0%					
					
Transport, Storage and Communication	775	15.0%	1.9%	4.6%	4.6%	7.6%	1.9%					

There was a significant difference (F statistic = 72.531, D.F. = 6, *p *< 0.0001) in ages by industry, presented in Table 2, where the youngest population worked in the hotel and restaurants industry with a mean age of 31.2 and the oldest population worked in the electricity, gas and water sector (mean age 40.0). There were also significant differences in the proportion of males and females by industry (*χ*^2 ^= 411.9, d.f. = 6, *p *< 0.0001), job type (*χ*^2 ^= 736.5, d.f. = 6, *p *< 0.0001) and contract type (*χ*^2 ^= 891.6, d.f. = 6, *p *< 0.0001). The fishing industry, one of the largest employers in Namibia, had significantly fewer employees on permanent contracts and the vast majority working in labour jobs. The electricity, gas and water industry had the highest rate of sick days (1.2) followed by the fishing sector (1.1) while the agriculture sector reported the lowest number of sick days (0.5). The agriculture, fishing, electricity, gas and water, and wholesale and retail trade industries all had significantly higher sick day rates than the retail sector which was used as a reference group.

### Risk factors

The majority reported they had never smoked (82.1%, 95%CI: 79.8-81.6), almost one third of participants (27.0%) presented with an elevated or high blood pressure, many (39.2%) had a BMI of 25 or greater and 18.7% (95%CI: 17.8-19.6) had central obesity for their gender (Table 3).

**Table 3:** Distribution of findings from biomedical screening

Variable	Levels	n	%	95% CI
**Risk Factors**				

Blood Pressure				

	Normal (< 140/90 mm/Hg)	5,580	72.8%	71.8-73.8
	
	Elevated (140/90-153/103 mm/Hg)	1,301	17.0%	16.1-17.8
	
	High (≥ 154/104 mm/Hg)	767	10.0%	9.3-10.7

BMI				

	Underweight (< 18.5)	476	6.2%	5.7-6.7
	
	Normal (18.5-24.9)	4,160	54.3%	53.1-55.4
	
	Overweight (25-29.9)	1,851	24.1%	23.2-25.1
	
	Obese (≥ 30)	1,146	14.9%	14.1-15.7

Waist Circumference				

	Normal	6,195	80.8%	79.9-81.7
	
	Above limit	1,435	18.7%	17.8-19.6

Smoking behaviour				

	Smokes	1,346	17.6%	16.7-18.4
	
	Never smoked	6,186	80.7%	79.8-81.6

**Conditions and diseases**				

Hypertension				

	No	6,171	80.5%	79.6-81.4
	
	Yes	1,277	16.7%	15.8-17.5

Diabetes				

	No	6,788	88.5%	87.8-89.3
	
	Yes	119	1.6%	1.3-1.8

Haemoglobin				

	Normal	5,654	73.8%	72.8-74.7
	
	Anaemia	479	6.2%	5.7-6.8

HIV				

	Positive	694	9.1%	8.4-9.7
	
	Negative	6,057	79.0%	78.1-80.0

Hepatitis B				

	Positive	524	6.8%	6.3-7.4
	
	Negative	5,621	73.3%	72.3-74.3

Syphilis				

	Positive	88	1.1%	0.9-1.4
	
	Negative	6,074	79.2%	78.3-80.1

Analysis by industry shows that in terms of risk factors, the fishing, agriculture and electricity, gas and water industries all had a prevalence of employees with elevated or high blood pressure above 28%. The prevalence of elevated or high blood pressure was significantly different among industries (*χ*^2 ^= 100.0, d.f. = 6, *p *< 0.0001) The lowest prevalence of abnormal blood pressure was found in the wholesale and retail trade and repair of motor vehicles industry which was still 17.1% (Table 2). In addition, at least a quarter of employees in all industries were found to be overweight or obese. The majority of employees across all industries did report smoking, although the agriculture industry did report the highest prevalence of smokers at 34.8%.

### Conditions and diseases

The overall prevalence of HIV in participants was 9.1% (95%CI: 8.4-9.7). Prevalence of antibodies against hepatitis B surface antigen (HbsAg) was 6.8% (95%CI: 6.3-7.4) while prevalence of syphilis antibodies was 1.1% (95%CI: 0.9-1.4). In addition, 1.7% (95%CI: 1.0-2.4) had diabetes according to accepted guidelines [22].

There was more variation in conditions and disease prevalence between industries than those found for risk factors. The prevalence of hypertension ranged from 10.7% in the hotels and restaurants industry to 21.7% in the fishing industry. Similarly, the hotels and restaurants industry had the lowest prevalence of diabetes at just 0.7% compared to 3.6% at the electricity, gas and water industry (*χ*^2 ^= 30.1, d.f. = 6, *p *< 0.0001). The fishing industry also had a high prevalence of anemia at 10.2% and the highest prevalence of HIV at 14.3% compared to just 4.6% for the transport, storage and communication industry. Prevalence rates of HIV varied significantly between industries (*χ*^2 ^= 125.1, d.f. = 6, *p *< 0.001). The prevalence of hepatitis B antigen was also above 7% for all sectors except for fishing and the hotels and restaurants industries. The prevalence of syphilis antigen was relatively low, although the agriculture sector did have a prevalence of 3.9%.

### Univariate analysis

Given the high variability of in demographic information, risk factors, and prevalence of conditions and diseases between industries, univariate analysis of independent variables to rate of sick days was conducted controlling for industry sector and are presented as Crude IRR (incidence rate ratio) in Table 4. For demographic information, having a part-time or fixed term contract was significantly associated with a reduction in sick days compared to those with permanent contracts. In addition working in the fishing sector (IRR = 1.53, 95%CI: 1.23-1.90), the services sector (IRR = 1.70, 95%CI: 1.32-2.20) or the wholesale sector (IRR = 1.39, 95%CI: 1.07-1.79) were significantly associated with a higher rate of sick days, while those in the agriculture sector had significantly less sick days (IRR = 0.66, 95%CI: 0.46-0.94) compared to retail, the reference group.

**Table 4:** Results of univariate and expanded models where the outcome is self-reported health-related absence from work in the previous 90 days

Demographic Information		Crude Rate Ratio (IRR)	Crude IRR 95% CI	*p*-value	Adjusted Rate Ratio (IRR)	Adjusted IRR 95% CI	*p*-value
Industry				< 0.0003			
			
	Retail and Trade						
				
	Agriculture	0.66	(0.46-0.94)	0.02			
				
	Fishing	1.53	(1.23-1.90)	< 0.01			
				
	Electricity, gas & water	1.70	(1.32-2.20)	< 0.01			
				
	Wholesale trade and repair of motor vehicles	1.39	(1.07-1.79)	0.01			
				
	Hotels and Restaurants	1.01	(0.66-1.55)	0.96			
				
	Transport, Storage and Communication	1.20	(0.90-1.59)	0.21			
			
Contract Type							
			
	Permanent						
				
	Part-time/Short-term	0.74	(0.63-0.89)	< 0.001			
			
Job Type							
			
	Labour						
				
	Administration Work	1.03	(0.85-1.24)	0.80			
			
Age							
			
	Age	1.00	(0.99-1.01)	0.97			
			
Sex							
			
	Male						
				
	Female	1.07	(0.90-1.26)	0.41			
	
		**Crude Rate**	**Crude IRR**			**Adjusted Rate**	**Adjusted IRR**

**Risk Factors**		**Ratio (IRR)**	**95% CI**	***p*-value**	**Ratio (IRR)**	**95% CI**	***p*-value**

BMI							
	BMI z-score	1.04	(0.96-1.13)	0.32	0.98	(0.90-1.07)	0.72

Waist Circumference							

	Normal						

	Above limit	1.21	(0.99-1.48)	0.06	1.10	(0.90-1.34)	0.42

Blood Pressure							

	Normal (< 140/90 mm/Hg)						
	
	Elevated (140/90-153/103 mm/Hg)	0.75	(0.60-0.92)	0.01	0.74	(0.59-0.91)	0.01
	
	High (≥ 154/104 mm/Hg)	1.10	(0.84-1.43)	0.50	1.03	(0.78-1.35)	0.85

Smoking Status							

	Never smoked						

	Smoker	1.09	(0.89-1.34)	0.40	1.15	(0.94-1.42)	0.18

Hypertension							

	No						
	
	Yes	1.14	(0.92-1.41)	0.22	1.07	(0.86-1.34)	0.55

Diabetes							
	No						
	
	Yes	3.40	(1.91-6.04)	< 0.01	3.67	(2.06-6.55)	< 0.01

Haemoglobin							
	Normal						
	
	Anaemia	1.82	(1.35-2.46)	< 0.01	1.59	(1.17-2.18)	< 0.01

HIV							
	Negative						
	
	Positive	1.55	(1.18-2.04)	< 0.01	1.47	(1.12-1.95)	0.01

Hepatitis B							
	Negative						
	
	Positive	1.01	(0.75-1.37)	0.92	1.14	(0.85-1.55)	0.38

Syphilis							
	Negative						
	
	Positive	0.64	(0.31-1.37)	0.22	0.83	(0.40-1.69)	0.60

*adjusted IRR were calculated after controlling for industry, age, sex, job status, and contract type. All IRR were calculated by exponentiating the beta coefficients obtained from negative binomial regression

No risk factor had a significant association with an increase in sick days. However, there were associations with respect to conditions and disease. Diabetes had the highest effect size on the rate of sick days (IRR = 3.40, 95%CI: 1.91-6.04). HIV infection was also associated with a significant increase in sick days (IRR = 1.55, 95%CI: 1.19-1.74) as was anaemia (IRR:1.82, 95%CI: 1.21-2.23).

### Expanded models

Analysis of all health screenings were remodeled controlling for industry, sex, age, job status, and contract type. Results of these expanded models are also presented in Table 2 as Adjusted IRR (incidence rate ratio). None of the covariates were found to be significant effect modifiers, as most of the adjusted incidence rate ratios remained close to the values found in univariate analysis. Diabetes, elevated blood pressure, anaemia and being HIV positive were the only conditions significantly associated with absenteeism.

## Discussion

This paper describes the association of multiple health determinants with an important labor productivity variable (absenteeism) in an African context. The industries represented are similar to those of the formal private sector of Namibia, although the sample is not representative of the entire formal sector of the country. Agriculture is the largest industry in Namibia (15.9%), followed by employment in private households (10.9%) (not captured in this study) and retail (9.1%) which was the largest group represented by this study [23]. Other sectors not represented in this study include public administration, education, mining and real estate. However, other important industries such as services, retail, tourism, fishing, and agriculture were included. The distribution of participants among different sectors does not reflect the proportions present in the Namibian formal sector, but does cover a large range of activities.

Namibia enacted the Labour Act in 1992, which was revised in 2007 (Act 11, 2007). Both the old and new Labour Act gives all employees (permanent and short-term contracts) a right to sick leave. The Labour Act states that during the first 12 months of employment, employees who work 5 days per week accrue 1 day of sick leave for every 5 weeks of employment. Employees that work 5 days per week are entitled to 30 working days of sick leave and those working 6 days per week can take up to 36 working days of sick leave, in a 3 year cycle. While the law may not be applied consistently everywhere, the majority of companies comply in providing paid sick leave [24]. A medical certificate is also required for sick leave, which may act as a barrier to some seeking care given that they may have to pay out-of-pocket. In addition, employers are required to pay employees full salary benefits for the allowed sick leave. However, there are no studies published looking at employer compliance with the law and the effect on employee absenteeism in Namibia. It is possible that employees may not use sick leave if they know they will not be compensated or that there may be some threat to their position from several absences.

There are a number of reports examining absenteeism rates in Europe and the United States. These reports show average sick leave rates ranging from 5.1 days per year for employees in Europe to just over 8 days per year for employees in the United States and up to 12 days per year for public sector employees in those countries [25,26]. Assuming the same rates of absenteeism in this study hold for the full year, we could expect there to be an average rate of sick leaves taken by the study participants close to 4 days per year. This calculation would put Namibian absenteeism rates in the formal sector somewhat below the estimates from the United States and Europe. Similar statistics for neighboring sub-Saharan African countries were not readily accessible. Despite much progress toward strengthening the health system, access to care for Namibians remains an area of concern. Public health facilities, which are the most accessible, are understaffed and patients may have to pay out-of-pocket [27]. These barriers to access may also be reducing absenteeism as employees decide to continue working rather than trying to seek care.

Compared with current research on absenteeism in the workplace, this study looks at a variety of health-related determinants without focusing on any one condition. It provides some insight into the prevalence of different risk factors and conditions among workers in various industries as well as the relative impact of those factors on absenteeism. The majority of absenteeism research is focused on high-income countries [11-14], and highlights the effect of particular chronic conditions on absenteeism. The findings of these studies consistently find a strong association between NCDs, their risk factors, and increased absenteeism. For most absenteeism-related studies in high-income countries, the recall period is longer and can be validated against employee records which help minimize recall bias. This level of information is not available for the current study as with many studies looking at sickness work absence in a sub-Saharan context [9,15-17]. As a result, the true level of absenteeism in this population may be underestimated as a result of recall bias or may be subject to whether employees have access to basic care services provided by some of the employers, as with the agriculture sector [10].

It is apparent from findings in Table 3 that NCDs and their associated risk factors are affecting the working population in Namibia. This is consistent with findings from studies in high-income countries [11,12]. This survey found a relatively high proportion of central obesity (18.7%) and elevated or high blood pressure (27.0%). In addition, diabetes had a significant impact on the rate of sick days although affecting a relatively low proportion of the population (1.7%). Moreover, anaemia which may be caused by a large number of conditions, including HIV, was also found to affect absenteeism. The mix of NCD and communicable disease is in line with the epidemiological transition that many low- and middle-income countries face today [28].

The most significant finding of this study is the association of diabetes with absenteeism. Random blood glucose is not a rigorous test for conditions like impaired glucose tolerance or diabetes and is sensitive to whether a person has eaten recently. However, the American Diabetes Association has established that a random blood glucose measurement above 11.1 mmol/L is very likely a sign of diabetes [22]. Therefore, although the actual diagnosis of diabetes could not be made, the current glucose results suggest that diabetes has an important impact on absenteeism and employee health.

Almost half of those who were overweight or obese also had high blood pressure readings and elevated blood glucose. This finding is compatible with other studies that have shown that the majority of people diagnosed with one non-communicable disease have co-morbidity with another [28,29]. NCDs share many common risk factors and several of those are modifiable, especially through lifestyle changes. These shared factors provide an opportunity for prevention strategies centered on improving diet, physical activity, and smoking reduction. Education strategies to reduce these risks could be included in workplace wellness programs and could have an effect on both the health of employees and reducing absenteeism.

Despite showing a less significant impact on absenteeism in this study, HIV is still a significant determinant of increased sick days. In the current study it is not possible to determine reasons for sick leave or to directly relate absenteeism to HIV. It is possible that some employees missed work in order to access ART care in a location away from their workplace. Conversely, it is possible that a number of the HIV positive workers are still in the early phase of infection and therefore are not yet on ART. These people could have more frequent (short) episodes of sickness, as compared to HIV negative people or HIV positive people on ART. HIV positive workers could also have other HIV positive family members and take more frequent sick leave days to care for them.

Discovering the reasons behind diabetes- and HIV-related absenteeism may help the private sector provide services that minimize costs lost to employee sickness. For the fishing sector, prevalence of HIV appeared much higher than the average for the whole population (14.3% compared to 9.0%, respectively). Where HIV/AIDS is more prevalent in certain sectors, special attention should be given to providing adequate services such as prevention education and ART. The lower relative impact of HIV/AIDS compared to diabetes may be a result of successful intervention programs already in place among employers, including the exemplary high ART coverage achieved in Namibia [30].

The impact of conditions like hepatitis B and syphilis were negligible and did not contribute to increased rates of sick days in the short-term. Prevalence estimates for hepatitis B surface antigen were below those reported for sub-Saharan Africa (> 8%) [3]. The same was true for syphilis, which is well below average rates reported in general population surveys in Africa [3]. Given the natural progression of these diseases in the absence of treatment, it is possible that infection could lead to long-term disability and contribute to future loss in overall productivity for companies with a large proportion of affected employees [24].

The fishing, services, and wholesale sectors had significantly higher rates of sick days when compared to the largest sector in this study, retail, even after controlling for other factors. The prevalence of HIV and diabetes were some of the highest in these sectors as well, which may indicate causal relationships. Conversely, the agricultural sector had significantly less absenteeism, perhaps because a number of farms in Namibia provide health-related services on site to their employees [20].

All of the above results are possibly subject to a bias toward workers who are fit enough to attend work on the day of the screening. If this is the case, the effects reported here are an underestimate of the true impact of these and other health factors on sick days for workers in Namibia.

### Limitations

Because this is a cross-sectional survey, it is impossible to determine causation and only associations between independent variables and the outcome (absenteeism) can be ascertained. In addition, the survey was meant as an awareness and management information exercise for companies and was not powered to detect particular associations. This increases the potential for type II statistical errors or the probability of finding a significant result when one does not actually exist. Because participation was voluntary, there may be a selection bias which would make results not applicable to the general population of employees. For the outcome variable (absenteeism), 890 people were missing data or refused to answer the question, which could bias the results as well. It could also be that HIV positive people preferentially declined to participate in the HIV test. No particular adjustments were made for this missing data as this is a cross-sectional survey and missing data were not found to be systematically distributed throughout the sample of industries. None of the variables had a greater than 5% proportion of missing data. While this survey collected information on those who refused to learn their results, it did not provide information on those who refused to have the test done at all.

Finally, it should be stated that the primary outcome variable (sick days) was not verified against employer records and is subject to recall bias as with any self-reported information. This bias was minimized by using a relatively short recall period of 90 days. Therefore, results cannot be extrapolated to longer time periods of observation. In addition, an important factor that could influence results, are the companies' policies regarding sick leave. These were not reviewed in the current study, but more liberal leave policies might result in higher reported absenteeism. There is a risk of underreporting of absenteeism due to cultural or workplace pressure which is not reflected in the findings. We did not review the workplace policies of each of the companies for this study, which would help to expand on this point, as it was beyond the capacity of this analysis to do so. However, this would be an important aspect to consider in the future.

There is no information available on expected absentee rates for employees in the Namibian formal sector which makes comparison of these results to a baseline impossible. However, the findings of this study may help to prioritize areas of intervention for health of employees including risk factors related to NCDs and HIV.

## Conclusion

While significant gains have been made in the provision of treatment for HIV/AIDS in Namibia, it is still a significant factor contributing to absenteeism among workers. In addition, this study shows non-communicable disease factors that are significantly contributing to increases in sick days, especially high blood glucose and high blood pressure. Wellness and healthy living education programs have been shown to have significant impacts on the incidence of chronic conditions in high-income countries [25,31]. Similar approaches could be developed and tested for the unique needs of this low- and middle-income country setting.

As other studies have shown that factors related to NCDs have an impact on employee productivity and absenteeism, companies should consider providing education and basic screening services related to healthy living and risk-reduction for NCDs. The impact of chronic infection with other conditions such as hepatitis B and syphilis proved less pronounced in Namibia. Company-provided services to reduce absenteeism should focus on the full spectrum of HIV prevention, treatment and care, as well as wellness education for risk factors related to NCDs including healthy eating and exercise. These recommended interventions should be implemented with simultaneous cost-effectiveness analyses, including the costing of absenteeism, to assess which interventions may be the most effective. If companies were to purchase insurance for their employees, or provide wellness services, these should include both HIV-related prevention and treatment as well as information on the risks and management of NCDs.

## Competing interests

Leonor Guariguata is a consultant to the PharmAccess Foundation and a full time employee of the International Diabetes Federation.

Ingrid de Beer is a full time employee of the PharmAccess Foundation in Namibia.

Rina Hough is a full time employee of the PharmAccess Foundation in Namibia.

Els Bindels is a full time employee of the PharmAccess Foundation in Namibia.

Delia Weimers-Maasdorp is a full time employee of the PharmAccess Foundation.

Frank Feeley is professor of international health at the Boston University School of Public Health.

Tobias Rinke de Wit is an employee of the PharmAccess Foundation in the Netherlands.

## Authors' contributions

LG - conducted the statistical analysis and wrote the manuscript.

IDB - contributed to the design and conduct of the study as well as providing information on the Namibian formal sector.

RH - conducted the data collection, cleaning, and primary descriptive analyses. DWM - designed the survey instrument and conducted the survey.

FF - contributed to the analysis with methods on occupational health research and revised the manuscript.

TFRDW - project lead and contributed to the writing, review, and interpretation of findings of the analysis. All authors read and approved the final manuscript.

## Funding sources

The funding for the Bophelo! Project originated from both the public sector (60% of all costs are funded through international donors, more information at http://www.pharmaccess.org) and the domestic private sector (employers paid part of the costs at each screening site). The Bophelo! Project is a partnership between the PharmAccess Foundation, the Namibian Business Coalition on AIDS, and the Namibian Institute of Pathology, supported by the Minsitry of Health and Social Services. The project was initially created using funds from the Dutch Postcode Loterij, through the Dutch Aidsfonds, Stop AIDS Now!, HIVOS and the Global Fund to fight AIDS, TB and Malaria.

## Disclosures

The authors have no conflicts of interest to disclose.

## Pre-publication history

The pre-publication history for this paper can be accessed here:

http://www.biomedcentral.com/1471-2458/12/44/prepub
